# Simulation of pedestrian evacuation route choice using social force model in large-scale public space: Comparison of five evacuation strategies

**DOI:** 10.1371/journal.pone.0221872

**Published:** 2019-09-06

**Authors:** Jibiao Zhou, Yanyong Guo, Sheng Dong, Minjie Zhang, Tianqi Mao

**Affiliations:** 1 School of Civil and Transportation Engineering, Ningbo University of Technology, Ningbo, China; 2 College of Transportation Engineering, Tongji University, Shanghai, China; 3 Jiangsu Key Laboratory of Urban ITS, Southeast University, Nanjing, China; Beihang University, CHINA

## Abstract

The primary objective of this study is to compare pedestrian evacuation strategies in the large-scale public space (LPS) using microscopic model. Data were collected by video recording from Tian-yi square for 36 hours in city of Ningbo, China. A pedestrian evacuation simulation model was developed based on the social force model (SFM). The simulation model parameters, such as reaction time, elasticity coefficient, sliding coefficient, et al, were calibrated using the real data extracted from the video. Five evacuation strategies, strategy 1 (S1) to strategy 5 (S5) involving distance, density and capacity factors were simulated and compared by indicators of evacuation time and channel utilization rate, as well as the evacuation efficiency. The simulation model parameters calibration results showed that a) the pedestrians walking speed is 1.0 ~ 1.5m/s; b) the pedestrians walking diameter is 0.3 ~ 0.4m; c) the frequency of pedestrian arrival and departure followed multi-normal distribution. The simulation results showed that, (a) in terms of total evacuation time, the performance of S4 and S5 which considering the capacity and density factors were best in all evacuation scenarios, the performance of S3 which only considering the density factor was the worst, relatively, and S1 and S2 which considering the distance factor were in the middle. (b) the utilization rate of channels under S5 strategy was better than other strategies, which performs best in the balance of evacuation. S3 strategy was the worst, and S1, S2 and S4 were in the middle. (c) in terms of the evacuation efficiency, when the number of evacuees is within 2, 500 peds, the S1 and S2 strategy which considering the distance factor have best evacuation efficiency than other strategies. And when the number of evacuees is above 2, 500 peds, the S4 and S5 strategy which considering the capacity factor are better than others.

## Introduction

With the continuous expansion of the city scale and the rapid growth of the urban population, people’s social and economic activities and cultural exchange activities are becoming more frequent. However, due to large-scale gatherings and unpredictable situations, numerous casualties and/or stampedes often occur [1, 2]. According to statistics from Wikipedia, more than 100 human stampedes have occurred in the world and 9,837 people were killed since the 1980s [3]. For instance, in April 15, 1989, a total of 766 people, almost all Liverpool supporters, were injured and 96 people died in the disaster in Hillsborough Stadium, Sheffield, UK [4]. Since 1990s, the location of human stampedes and crushes began to show a trend of diversification [4–6], such as at stations, stadiums, festival venues, concert halls, shopping venues, and even schools. For example, in August 31, 2005, at least 950 people were killed in the Baghdad bridge stampede. In December 31, 2014, 36 people died and 42 people were injured due to a stampede during New Year’s celebrations at the Bund in Shanghai, China [7]. The same tragedy continues with major events, for instance, in June 3, 2017, one person was killed and more than 1,500 others were injured when panic erupted during a screening of the UEFA Champions League Final in Turin, Italy [8]. In December 8, 2018, six people were killed and dozens more injured in a human stampede as frantic concertgoers tried to exit a packed Lanterna Azzurra club in Corinaldo, Italy [9]. The human stampedes resulted from the large-scale activates bring huge economic losses to the society.

Therefore, a better understanding of pedestrian evacuation route can be beneficial of preventing such stampedes and reducing the injury and death. Firstly, the passway for escaping in the large-scale public space (LPS) is limited, therefore, the evacuation route choice of pedestrians is also limited. Secondly, pedestrian evacuation route selection is affected by many factors, such as evacuation channel characteristics, the width/length of evacuation route, capacity of evacuation route, personal characteristics, and so on. Thirdly, the evacuation drills in LPS require a lot of human, material and financial resources, and it is difficult to repeat training many times in the evacuation drills. With the development of computer technology, traffic simulation technology [10–12] has been widely used in many fields, such as military, industrial, transportation, logistics, safety assessment, and traffic conflicts, because of its advantages of less consumable, repeatable and non-dangerous. It provides a new idea for traffic evacuation simulation, especially for pedestrian evacuation route selection. Hence, the objective of this study is to compare five pedestrian evacuation strategies in the large-scale public space (LPS). A simulation model was developed and calibrated using the video data from Tian-yi square in city of Ningbo, China. The measures of evacuation time and channel utilization rate for each evacuation strategy were compared to evaluate the strategies.

## Literatures review

### Influence factors on evacuation route choice

Understanding the influence factors on pedestrian evacuation route choice is essential in the planning and the design of large-scale public spaces, such as stadiums, airports, railway stations, and bus terminals. Like most walking processes, the evacuation route choice strategies are largely subconscious and affected by local and global factors [13–16].

Previous studies showed that numerous factors such as pedestrian emotion (pleasantness, fear, and anger), crowd degree, cooperation and selfish behavior, conformity behavior, route capacity and length, illumination condition, barrier, route familiarity, guidance sign, and exits visible were important attributes to pedestrians’ rout choice [13–16]. For instance, Hoogendoorn et al. [13] indicated that the route directness pertains not only to the route length, but also to its complexity. Pedestrians appear to frequently choose the shortest route, albeit they are seldom aware that they are minimizing distance as a primary strategy in route choice. Kinateder et al. [14] also found that exit choice in an emergency evacuation scenario was influenced by exit familiarity and neighbor behavior.

Other studies indicate that besides distance, illumination condition, route distance, guidance sign, pleasantness and route familiarity et al. are important route attributes together producing a high correlation with the route preferences, and the influence effects of above factors were not the same under different facility conditions [13–19]. For example, in a classroom, Chen L, Tang T, Huang H, et al. [15–19] found that the position, congestion, group behavior and backtracking behavior have significant effects on children’s route choice, but gender and guidance have no prominent impacts on children’s route choice. Guo R, Huang H, and Wong [20] revealed several typical forms of behavior related to preference for destination, effect of capacity, interaction between pedestrians, following behavior and evacuation efficiency. In addition, several studies indicated that habit, number of crossings, pollution and noise level, safety and shelter from poor weather conditions, and stimulation of the environment were also important factors in pedestrian route choice behavior [21–23].

### Social force model for pedestrian simulation

To well understand pedestrian crowd behavior and evacuation behavior, microscopic simulation models have been developed in recent decades, in which a single individual’s behavioral and its interaction with other individuals as well as the environment can be modeled and simulated. A well-known model to simulate pedestrian crowd or evacuation behavior is called the social force model, a typically physical-force based model, which was initially innovated by Dirk Helbing et al. [1, 24, 25]. Generally speaking, their greatest contribution was in human behavior, namely, they put the rules of pedestrian behavior into an equation of motion. This model is for pedestrian dynamics, has been widely accepted [24–26] and mainly used in the description of pedestrian behavior, pedestrian simulation of crowd evacuation. Interestingly, in this model, certain psychological factors are abstracted into SFM parameters such as “desired velocity”, “repulsive force”, “attraction force”, and “social force”. To some extent, these concepts or parameters are not physical entities since they only describe human behavioral changes and movements. Thus, the model is not typically within the scope of physics study, but in an interdisciplinary domain. In brief, the social force model exhibits a bridge between the physics laws and psychological principles regarding crowd motion.

With the continuous expansion of the application field of SFM, a review of the literatures regarding SFM was found in many studies [24–26]. Particularly, the SFM has been widely applied in simulation of crowd evacuation in the past two decades. For example, instead of a fluid-dynamic model, Dirk Helbing et al. [24–26] used the social force model to simulate the crowd dynamics of pedestrians in normal and evacuation situations. It was found that the proposed model (SFM) could not only be applied to test buildings for their suitability in normal situations, but also in emergency situations. Meanwhile, Dirk Helbing et al. [24–26] proved that the social force model was capable of describing the self-organization of several observed collective effects of pedestrian behavior very realistically. Porter et al. [27] presented an integrated modeling framework to capture pedestrian walking behavior in congested and uncongested conditions, which the framework was built using a combination of concepts from the social force model. In addition, the robust model was also applied to provide valuable insights into simulate the dynamical features of escape panic [1, 21, 22], abnormal behaviors localization [28], exit-selecting behaviors analysis [29], detour behavior description [30], exit assignment strategy [31], pedestrian behavior analysis [32–37], and pedestrian traffic [38]. The main contribution of this study is the development of a pedestrian evacuation route choice model based on social force theory, which enables to consider the characteristics of pedestrian behavior in the large-scale public space (LPS). Furthermore, an estimation approach for calibrating the social force model based on real arrival-departure data is proposed. Last, the validation is conducted to confirm the model performance in terms of evacuation strategy such as evacuation time, channel utilization rate, as well as the evacuation efficiency.

## Data collection

### Study location

The IRB (institutional review board) of Academic Committee of Ningbo University of Technology waived the need for consent from the participants. Data were collected at the Tian-yi square, which is the largest outdoor large-scale public space (LPS) in city of Ningbo, China. It is an open shopping plaza in the form of shopping, tourism, leisure, entertainment, sports and food, which is a representative and typical LPS in China. The total construction area of Tian-yi square is 220,000 m^2^, the central square area of 35,000 m^2^, including 10,000 m^2^ of landscape water and 30,000 m^2^ of green [39]. Due to its commercial and entertainment features, it attracts a large number of tourists every day.

The LPS has eight evacuation channels. Data collection process in this study was divided into two parts: (a) manual investigation to collect the LPS’s information, including the width of channel and the distance interval between the two adjacent channels, and (b) video recording to collect pedestrians’ arrivals and departures information, including the number of pedestrian arrivals, number of pedestrian departures, and total number of pedestrians in the LPS.

Eight high-resolution cameras were used at each channel. Position of each camera was placed on a temporary support, approximately 50 m upstream of the channel, angled across the channel. These cameras were intended to obtain macro parameters of pedestrian flow while approaching the channel, such as pedestrian flow, walking speed, and density. Channel width, channel length and channel spacing were collected at the same time.

In order to have enough sample size, videos were recorded from 08:00 a.m. to 20:00 p.m. at weekday and weekend for three days. A total of 12 hours × 3 days = 36 hours of video data was collected.

### Data results

**Fig 1:**
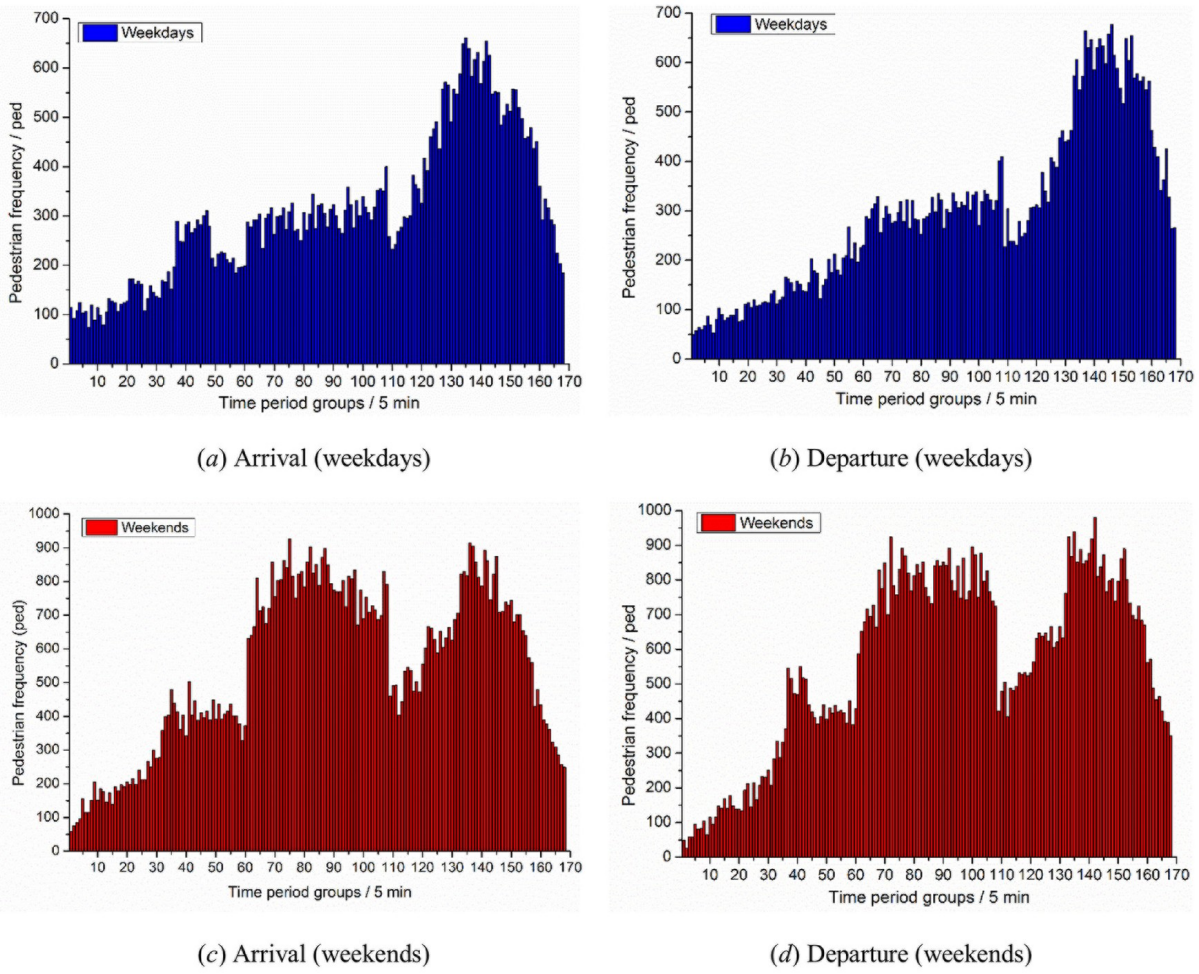
Results of pedestrian arrival-departure characteristics in the LPS.

1. Evacuation channel characteristics
  The pedestrian evacuation channel characteristics are as follows, the width of No.1- No.8 channels are 6.5 m, 11.5 m, 2.4 m, 3.0 m, 10.0 m, 8.6 m, 4.2 m, and 3.9 m, respectively. The distance interval from No.1 channel to No.8 channel are 100 m, 75 m, 80 m, 90 m, 150 m, 60 m, 50 m, and 30 m, respectively. These data are applied to pedestrian evacuation simulation model.
2. Pedestrians speed
  The survey result shows that the maximum pedestrian walking speed is 1.4m/s, the minimum walking speed is 0.7m/s, and the average value is 1.2m/s. In the pedestrian evacuation simulation model, the setting of walking speed is very important, which is a range type (requires a minimum value and a maximum value). The result of pedestrian speed in the LPS is used in the simulation model.
3. Pedestrian arrival-departure characteristics
  The survey time of the original data source is 8:00 ~ 22:00, 14 hours in total. The frequency of pedestrians entering and leaving Tian-yi Square is counted every 5 minutes as a time interval. A total of 672 sets of frequency data are obtained. The data results of pedestrian arrival-departure characteristics are shown in Fig 1(*a*)–1(*d*).

As shown in Fig 1(a)–1(d), the frequency distribution of pedestrians’ arrival and departure shows that, the pedestrian arrival and departure is in accordance with the multivariate normal distribution trend. According to the trend analysis in the Fig 1(a)–1(d), three time periods are selected by 8:00~13:00, 13:00~17:00, and 17:00~22:00. Next, we set the time period of three normal distribution trends to TIME _A, TIME_B, IEM_C, the parameters are calculated from data, and we also respectively perform K-S test on the normal distribution fitting in three time periods, the results of one-sample kolmogorov-smirnov test are showed is Tables 1 and 2.

**Table 1:** One-Sample Kolmogorov-Smirnov Test (Arrivals).

Time periods		TIME_A		TIME_B		TIME_C	
Type of day		weekend	weekday	weekend	weekday	weekend	weekday
Frequency of survey period group/5min		60	60	48	48	60	60
Normal parameters	Mean	289.783	176.167	779.333	304.625	610.017	449.683
	Std. Deviation	123.520	65.633	71.154	31.075	175.354	134.641
Most Extreme Differences	Absolute	0.170	0.125	0.089	0.079	0.070	0.115
	Positive	0.147	0.125	0.077	0.079	0.050	0.100
	Negative	-0.170	-0.084	-0.089	-0.058	-0.070	-0.115
Kolmogorov-Smirnov Z		1.317	0.966	0.614	0.548	0.539	0.892
Asymp. Sig. (2-tailed)		0.062	0.309	0.845	0.925	0.933	0.405

**Table 2:** One-Sample Kolmogorov-Smirnov Test (Departures).

Time periods		TIME_A		TIME_B		TIME_C	
Type of day		weekend	weekday	weekend	weekday	weekend	weekday
Frequency of survey period group/5min		60	60	48	48	60	60
Normal Parameters	Mean	277.050	134.567	792.104	307.792	663.917	459.050
	Std. Deviation	155.993	52.466	72.473	31.014	168.471	144.764
Most Extreme Differences	Absolute	0.151	0.093	0.103	0.100	0.100	0.157
	Positive	0.129	0.093	0.055	0.100	0.098	0.107
	Negative	-0.151	-0.067	-0.103	-0.052	-0.100	-0.157
Kolmogorov-Smirnov Z		1.173	0.718	0.714	0.696	0.776	1.216
Asymp. Sig. (2-tailed)		0.127	0.681	0.688	0.718	0.584	0.104

The results of K-S test showed that,

1. The values of two-tailed asymptotic significance among TIME_A, TIME_B and TIEM_C are 0.062, 0.845 and 0.933 in frequency distribution test of pedestrian’s arrivals on weekends, which are higher than the critical value of 0.05. Therefore, the original hypothesis is valid, indicating that the frequency distribution of pedestrians’ arrival on weekends conforms to the normal distribution.
2. The values of two-tailed asymptotic significance among TIME_A, TIME_B and TIEM_C are 0.127, 0.688 and 0.584 in frequency distribution test of pedestrian’s departures on weekends, which are higher than the critical value of 0.05. Therefore, the original hypothesis is valid, indicating that the frequency distribution of pedestrians’ departures on weekends conforms to the normal distribution.
3. The values of two-tailed asymptotic significance among TIME_A, TIME_B and TIEM_C are 0.309, 0.925 and 0.405 in frequency distribution test of pedestrian’s arrivals on weekdays, which are higher than the critical value of 0.05. Therefore, the original hypothesis is valid, indicating that the frequency distribution of pedestrians’ arrivals on weekdays conforms to the normal distribution.
4. The values of two-tailed asymptotic significance among TIME_A, TIME_B and TIEM_C are 0.681, 0.718 and 0.104 in frequency distribution test of pedestrian’s departures on weekdays, which are higher than the critical value of 0.05. Therefore, the original hypothesis is valid, indicating that the frequency distribution of pedestrians’ departures on weekdays conforms to the normal distribution.

In probability theory, the normal distribution is a very common continuous probability distribution, and which is also useful because of the central limit theorem. Hence, in our experiment, we want to find the pedestrian arrival-departure characteristics through the probability density of the normal distribution with the survey data.

The probability density of the normal distribution is shown in Eq (1).

$$f(x)=\frac{1}{\sigma \sqrt{2\pi }}\text{exp}(-\frac{{\left(x-\mu \right)}^{2}}{2\sigma^{2}})$$ (1)

where x is the pedestrian frequency of entering and leaving the square in the unit time period (5min), μ is the mean or expectation of the distribution, σ is the standard deviation, and σ2 is the variance.

On weekends, the probability density function of pedestrians arriving at the square is shown in Eq (2).

$$f_{a}(x)=\left\{ \begin{array}{l} \frac{1}{123.520\times \sqrt{2\pi }}\text{exp}(-\frac{{\left(x-289.783\right)}^{2}}{2\times 123.520^{2}}),x\in the\;frequency\;of\;\text{TIME}\_A\\ \frac{1}{71.154\times \sqrt{2\pi }}\text{exp}(-\frac{{\left(x-799.333\right)}^{2}}{2\times 71.154^{2}}),x\in the\;frequency\;of\;\text{TIME}\_B\\ \frac{1}{175.354\times \sqrt{2\pi }}\text{exp}(-\frac{{\left(x-610.017\right)}^{2}}{2\times 175.354^{2}}),x\in the\;frequency\;of\;\text{TIME}\_C \end{array}\right.$$ (2)

On weekends, the probability density function of pedestrians leaving the square is shown in Eq (3).

$$f_{b}(x)=\left\{ \begin{array}{l} \frac{1}{155.993\times \sqrt{2\pi }}\text{exp}(-\frac{{\left(x-277.050\right)}^{2}}{2\times 155.993^{2}}),x\in the\;frequency\;of\;\text{TIME}\_A\\ \frac{1}{72.473\times \sqrt{2\pi }}\text{exp}(-\frac{{\left(x-792.104\right)}^{2}}{2\times 72.473^{2}}),x\in the\;frequency\;of\;\text{TIME}\_B\\ \frac{1}{168.471\times \sqrt{2\pi }}\text{exp}(-\frac{{\left(x-663.917\right)}^{2}}{2\times 168.471^{2}}),x\in the\;frequency\;of\;\text{TIME}\_C \end{array}\right.$$ (3)

On weekdays, the probability density function of pedestrians arriving at the square is shown in Eq (4).

$$f_{c}(x)=\left\{ \begin{array}{l} \frac{1}{65.633\times \sqrt{2\pi }}\text{exp}(-\frac{{\left(x-176.167\right)}^{2}}{2\times 65.633^{2}}),x\in the\;frequency\;of\;\text{TIME}\_A\\ \frac{1}{31.075\times \sqrt{2\pi }}\text{exp}(-\frac{{\left(x-304.625\right)}^{2}}{2\times 31.075^{2}}),x\in the\;frequency\;of\;\text{TIME}\_B\\ \frac{1}{134.641\times \sqrt{2\pi }}\text{exp}(-\frac{{\left(x-449.683\right)}^{2}}{2\times 134.641^{2}}),x\in the\;frequency\;of\;\text{TIME}\_C \end{array}\right.$$ (4)

On weekdays, the probability density function of pedestrians leaving the square is shown in Eq (5).

$$f_{d}(x)=\left\{ \begin{array}{l} \frac{1}{52.466\times \sqrt{2\pi }}\text{exp}(-\frac{{\left(x-134.567\right)}^{2}}{2\times 52.466^{2}}),x\in the\;frequency\;of\;\text{TIME}\_A\\ \frac{1}{31.014\times \sqrt{2\pi }}\text{exp}(-\frac{{\left(x-307.192\right)}^{2}}{2\times 31.014^{2}}),x\in the\;frequency\;of\;\text{TIME}\_B\\ \frac{1}{144.764\times \sqrt{2\pi }}\text{exp}(-\frac{{\left(x-459.050\right)}^{2}}{2\times 144.764^{2}}),x\in the\;frequency\;of\;\text{TIME}\_C \end{array}\right.$$ (5)

The parameters from Eqs (2)–(5) are used as input parameters in the simulation.

## Pedestrian evacuation simulation model

### Social force model (SFM) method

#### Principle of SFM

The social force model (SFM) is the most concerned pedestrian flow model so far [24–27, 31]. It is also one of the most widely used pedestrian traffic simulation models [10, 32–34]. The pedestrian database of simulation software used to simulate pedestrian flow is based on the SFM [24]. The SFM can simulate important phenomena of escape panic [1, 25, 28–30, 32–38], such as “clogging”, “faster is slower”, and “mass behavior”.

The basic principle of the social force model holds that the laws of pedestrian movement are affected by two factors: pedestrians’ own purpose and their surroundings. The former is an internal factor, and the latter an external factor. Both the factors constitute the driving force of pedestrian movement so that the pedestrian movement can be regarded as the result of the combined forces of the system. Drawing on the expressive formula of Newton’s Second Law,

F=m⋅a (6)

The social force can be described as a force that changes pedestrian movement. Therefore, the social force model establishes the tendency of pedestrian behaviors through the force analysis similar to Newton’s mechanics. The force to change pedestrian movement is a resultant force, which can be divided into three parts: self-driving force, resultant force among pedestrians, and resultant force between pedestrians and obstacles or boundaries. Its model expression is as follows,

$$\vec{f_{i}}(t)=m_{i}\frac{d\vec{v_{t}}}{dt}=\vec{f_{i}^{0}}(t)+\sum_{j\neq i}\vec{f_{ij}}+\sum_{w}\vec{f_{iw}}+\vec{\varphi_{i}}(t)$$ (7)

where $\vec{f_{i}}(t)$ is the resultant force on the pedestrian *i* at time *t*; $\vec{f_{i}^{0}}(t)$ is the self-driving force on the pedestrian *i* at time *t*; $\vec{f_{ij}}$ is the force on the pedestrian *i* acted by the pedestrian *j*; $\vec{f_{iw}}$ is the force on the pedestrian *i* acted by the obstacle or boundary *w*; and $\vec{\varphi_{i}}(t)$ is the force on the pedestrian *i* acted by other factors.

#### Self-driving force

The model expression of self-driving force is as follows,

$$\vec{f_{i}^{0}}(t)=m_{i}\frac{v_{i}^{0}(t)\vec{e_{i}^{0}}(t)-\vec{v_{i}}(t)}{\tau_{i}}$$ (8)

where $\vec{e_{i}^{0}}(t)$ is the expected movement direction of the pedestrian *i* at time *t*; $v_{i}^{0}(t)$ is the expected speed of the pedestrian *i* at time *t*; $\vec{v_{i}}(t)$ is the current speed of the pedestrian *i* at time *t*; *τ*_*i*_ is the reaction time of the pedestrian *i*; and *m*_*i*_ is the mass of the pedestrian *i*.

#### Force among pedestrians

The model expression of force among pedestrians is as follows,

$$\vec{f_{ij}}=\vec{f_{ij}^{soc}}+\vec{f_{ij}^{ph}}$$ (9)

$$\vec{f_{ij}^{soc}}=A_{i}\cdot \text{exp}\left\{\frac{r_{ij}-d_{ij}}{B_{i}}\right\}\cdot \vec{n_{ij}}$$ (10)

fijph→=Kθ(r−ijdij)nij→+kθ(r−ijdij)Δvjittij→ (11)

$$\Delta v_{ji}^{t}=(\vec{v_{j}}-\vec{v_{i}})\cdot \vec{t_{ij}}$$ (12)

θ(r−ijdij)={r−ijdij,(r−ijdij)>00,(r−ijdij)≤0 (13)

where $\vec{f_{ij}^{soc}}$ is the psychosocial force on the pedestrian *i* acted by the pedestrian *j*; $\vec{f_{ij}^{ph}}$ is the physical force on the pedestrian *i* acted by the pedestrian *j*; *A*_*i*_ is a constant parameter that characterizes the repulsive strength among the pedestrians; *B*_*i*_ is a constant that characterizes the range of the repulsion; *r*_*ij*_ is the sum of the radiuses of the pedestrians *i* and *j*; *d*_*ij*_ is the distance between pedestrians *i* and *j*; $\vec{t_{ij}}$ is the tangent direction perpendicular to the central connection on the pedestrians *i* and *j*; $\vec{n_{ij}}$ is the direction of the central connection on the pedestrians *i* and *j*; *K* is the elasticity coefficient of the bodies; *θ* is the piecewise function of *r*_*ij*_ − *d*_*ij*_; *k* is the sliding coefficient; and $\Delta v_{ji}^{t}$ is the speed difference between the pedestrians *i* and *j* in the tangent direction.

#### Force acted by the obstacles or boundaries

The model expression of force acted by the obstacles or boundaries is as follows,

$$\vec{f_{iw}}=\vec{f_{iw}^{soc}}+\vec{f_{iw}^{ph}}$$ (14)

$$\vec{f_{iw}^{soc}}=A_{w}\cdot \text{exp}\left\{\frac{r_{iw}-d_{iw}}{B_{w}}\right\}\cdot \vec{n_{iw}}$$ (15)

fijph→=Kθ(r−iwdiw)niw→+kθ(r−iwdiw)Δvwittiw→ (16)

θ(r−iwdiw)={r−iwdiw,(r−iwdiw)>00,(r−iwdiw)≤0 (17)

where $\vec{f_{iw}^{soc}}$ is the psychosocial force on the pedestrian *i* acted by the obstacle *w*; $\vec{f_{iw}^{ph}}$ is the physical force on the pedestrian *i* acted by the obstacle *w*; *A*_*w*_ is a constant parameter that characterizes the repulsive strength of the obstacle *w*; *B*_*w*_ is a constant that characterizes the range of repulsion; *r*_*iw*_ is the sum of the radiuses of the pedestrian *i* and the obstacle *w*; *d*_*iw*_ is the distance between the pedestrian *i* and the obstacle *w*; $\vec{t_{iw}}$ is the tangent direction perpendicular to the central connection on the pedestrian *i* and the obstacle *w*; $\vec{n_{iw}}$ is the direction of the central connection on the pedestrian *i* and the obstacle *w*; *θ* is the piecewise function of *r*_*iw*_ − *d*_*iw*_; and $\Delta v_{wi}^{t}$ is the speed difference of the pedestrian *i* and the obstacle *w* in the tangent direction.

### Pedestrian evacuation simulation process

**Fig 2:**
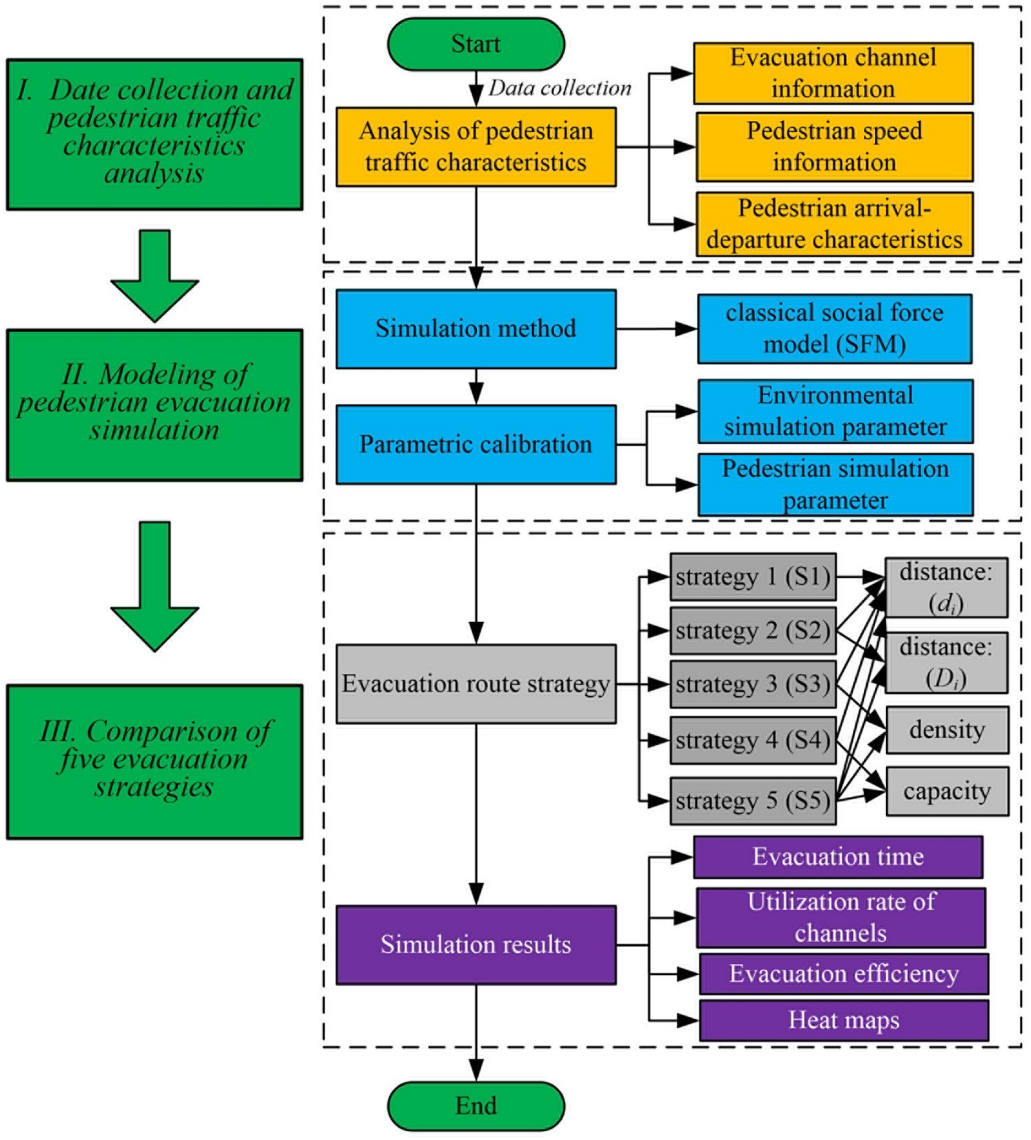
Simulation process of pedestrian evacuation.

In this study, the normal scene of crowd evacuation is simulated based on the SFM. The simulation process of pedestrian evacuation is as shown in Fig 2.

#### Pedestrian evacuation route choice

**Fig 3:**
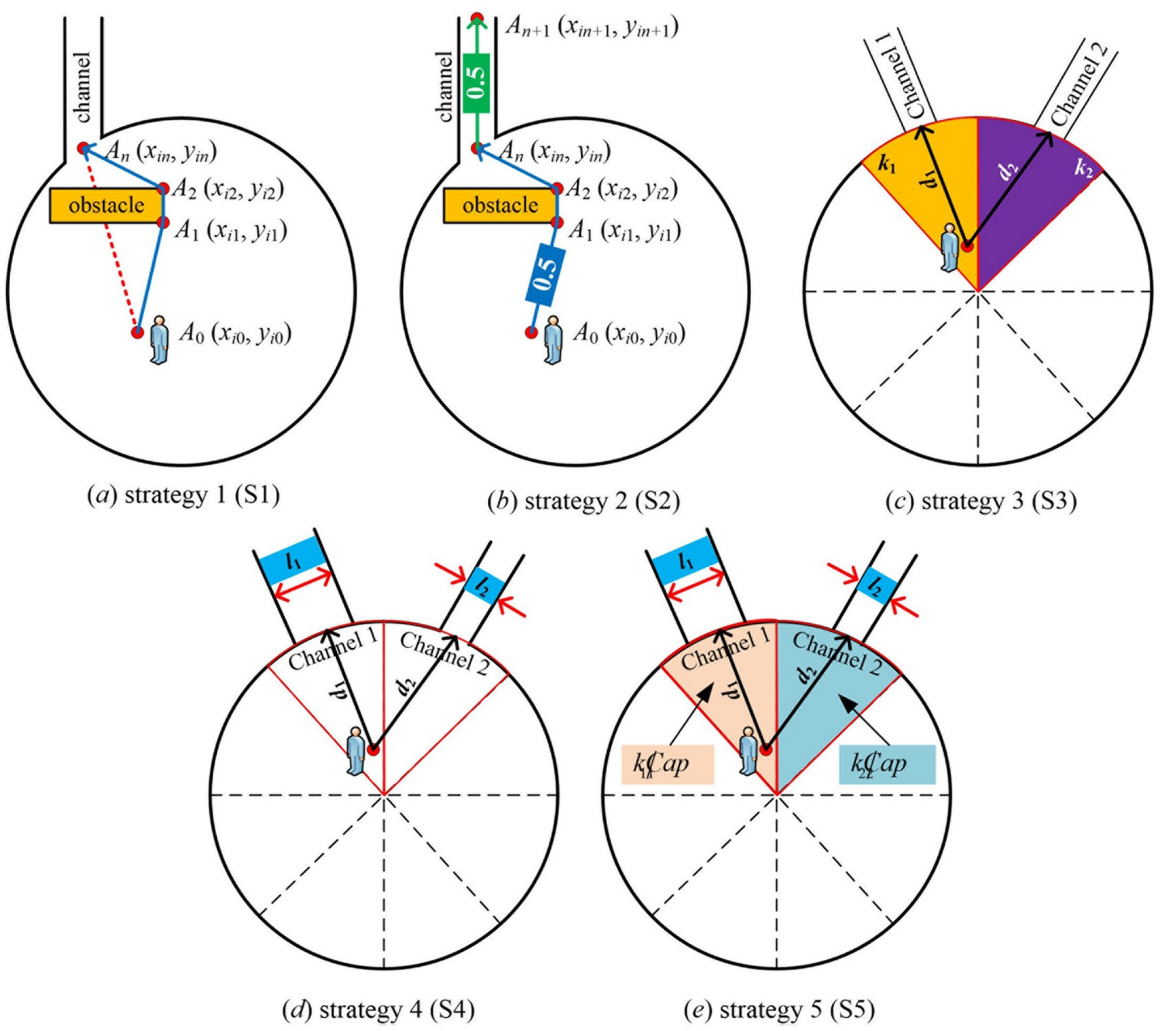
The illustrative diagram of five strategies.

Five pedestrian evacuation strategies were considered for route choice in the simulation. These evacuation strategies covered three factors (distance, density and capacity) that influence the evacuation performance. The structure of the five strategies is shown in Table 3 and Fig 3. In Table 3, the parameter *d*_*i*_ (meter as the unit) is the distance between one pedestrian’s location and the inner entrance of the channel *i*, *D*_*i*_ (meter as the unit) is the length of the channel *i*, *k*_*i*_ (*ped*/*m*^2^ as the unit) is the pedestrian density near the channel *i*, and *Cap*_*i*_ (*ped*/*s* as the unit) is the capacity of channel *i*. What’s more, *d*_*i*_ (meter as the unit) is the shortest distance when pedestrians consider obstacles in evacuation route choice, not the actual route length in their evacuation process. The detailed description is shown in the Fig 3.

**Table 3:** Five strategies.

Strategies	*d*_*i*_	*D*_*i*_	*k*_*i*_	*Cap*_*i*_
strategy 1 (S1)	√	×	×	×
strategy 2 (S2)	√	√	×	×
strategy 3 (S3)	√	×	√	×
strategy 4 (S4)	√	×	×	√
strategy 5 (S5)	√	√	√	√

#### Mathematical expressions of five strategies

1. Strategy 1: The shortest distance *d*_*i*_ between pedestrians’ location and the inner entrance of the channel *i* is the only considered factor.
  $$d_{i}=\sum_{j=0}^{n-1}\sqrt{{\left(x_{ij}-x_{i(j+1)}\right)}^{2}+{\left(y_{ij}-y_{i(j+1)}\right)}^{2}}$$ (18)
  where *A*_0_(*x*_*i*0_, *y*_*i*0_), *A*_*n*_(*x*_*in*_, *y*_*in*_) refers to pedestrians’ location at the beginning of evacuation process and the location of the entrance of the channel *i* respectively; *A*_*j*_(*x*_*ij*_, *y*_*ij*_) refers to the intersections of the shortest route for pedestrians to get around obstacles to the entrance of the channel *i* and obstacles.
2. Strategy 2: The whole shortest distance between of pedestrians’ location and the exit of the channel *i* is the main considered factor. The whole shortest distance is the sum of the shortest distance *d*_*i*_ between pedestrians’ location and the inner entrance of the channel *i* and *D*_*i*_, the length of the channel *i*.
  $$L_{i}=w_{1}\times d_{i}+w_{2}\times D_{i}$$ (19)
  where *L*_*i*_ is the whole shortest distance; *w*_1_ = *w*_2_ = 0.5 means factors such as congestion level and evacuation capacity of evacuation routes will not considered in strategy 2, and so the weights of *d*_*i*_ and *D*_*i*_ are the same.
3. Strategy 3: Considering the factors of pedestrian density *k* of the area around channels and distance *d*, that is, congestion level of evacuation routes is considered in evacuation route choice,
  $$f_{i}=w_{3}\times (1-\frac{k_{i}}{\sum_{j=1}^{n=8}k_{j}}\times \frac{d_{i}}{\sum_{j=1}^{n=8}d_{j}})+w_{4}\times (1-\frac{d_{i}}{\sum_{j=1}^{n=8}d_{j}})$$ (20)
  where *f*_*i*_ is the priority of channel *i*, and the final evacuation route is chosen as max{*f*_*i*_, *i* = 1, 2, ⋯, *n*}; *k*_*i*_ refers to the pedestrian density of the area around channel *i*; *w*_3_ and *w*_4_ are the weight of the density and the distance in evacuation route choice respectively, and their values are adjusted and optimized automatically according to the maximum priority in the simulation process to eliminate the contrary problem of routes selection to common sense. The simulation results show that when *w*_3_ = 0.7 and *w*_4_ = 0.3, the pedestrian evacuation route can be guaranteed. The area range is shown in Fig 3(c).
4. Strategy 4: Considering the factors of the channel capacity *Cap* and the distance *d*, that is, the evacuation capacity is mainly considered in evacuation route choice. This strategy can effectively avoid the possibility of all pedestrian choosing the same route in evacuation.
  $$g_{i}=w_{5}\times \frac{Cap_{i}}{\sum_{j=1}^{n=8}Cap_{j}}+w_{6}\times (1-\frac{d_{i}}{\sum_{j=1}^{n=8}d_{j}})$$ (21)
  $$Cap_{i}=3600\times l_{i}\times \frac{\Delta l}{\bar{v}}$$ (22)
  where *g*_*i*_ is the priority of channel *i*, and the final evacuation route is chosen as max(*g*_*i*_, *i* = 1, 2, ⋯, *n*); *Cap*_*i*_ refers to the evacuation capacity of the channel *i*; The simulation results show that when *w*_5_ = 0.8 and *w*_6_ = 0.2, the pedestrian evacuation route can be guaranteed. Δ*l* refers to the average longitudinal spacing in pedestrian evacuation; $\bar{v}$ refers to the average speed in pedestrian evacuation.
5. Strategy 5: The shortest distance *d*_*i*_, the channel length *D*_*i*_, the pedestrian density *k*_*i*_ and the capacity *Cap*_*i*_ are considered comprehensively. That is, when pedestrians make evacuation route choice, the congestion level and the evacuation capacity of routes are considered comprehensively:
  $$h_{i}=w_{7}\times (1-\frac{k_{i}/Cap_{i}}{\sum_{j=1}^{n=8}(k_{j}/Cap_{j})}\times \frac{d_{i}}{\sum_{j=1}^{n=8}d_{j}})+w_{8}\times (1-\frac{d_{i}}{\sum_{j=1}^{n=8}d_{j}})$$ (23)
  where *h*_*i*_ is the priority of channel *i*, and the final evacuation route is chosen as max(*h*_*i*_, *i* = 1, 2, ⋯, *n*).

### Parameter calibration of the SFM

#### Hypothesis of pedestrian simulation conditions

The following assumption is made for the simulation.

1. pedestrians: the special groups such as pregnant women and disabled people are ignored in this simulation.
2. channel: there are no obvious obstacles in the channel, the pedestrians’ sight is good, and the width of the channel is consistent.
3. simulation environment: the pedestrians’ activity area is limited to the open area of the square. Pedestrians’ entry and exit activities of the shops around the square are neglected. Pedestrians’ crossing is not allowed in landscape areas, pool areas, and green areas.
4. evacuation process: no dangerous incidents such as fatalities occur during the evacuation process. After the evacuation, the capacity of channel exit to the outside of square is infinite.
5. pedestrian psychology: the impact of pedestrian panic and conformity behaviors are ignored in the simulation.

#### Calibration of measurable parameters

For the environment parameters setting, it is scaled down in proportion according to the actual size of Tian-yi square. Next, the channel width and the channel length are calibrated by the actual, as shown in Table 4.

**Table 4:** Result of measurable parameters for calibration.

Parameters	Channels							
	No.1	No.2	No.3	No.4	No.5	No.6	No.7	No.8
width/m	6.5	11.5	2.4	3.0	10.0	8.6	4.2	3.9
length/m	36.0	35.0	33.5	34.0	33.0	31.0	44.0	38.0
interval/m	15.0	91.0	83.0	96.0	99.0	172.0	78.0	74.0
diameter/m	0.3~0.4	0.3~0.4	0.3~0.4	0.3~0.4	0.3~0.4	0.3~0.4	0.3~0.4	0.3~0.4
initial speed/m·s^-1^	0.3~0.7	0.3~0.7	0.3~0.7	0.3~0.7	0.3~0.7	0.3~0.7	0.3~0.7	0.3~0.7
comfortable speed/m·s^-1^	1.0~1.4	1.0~1.4	1.0~1.4	1.0~1.4	1.0~1.4	1.0~1.4	1.0~1.4	1.0~1.4
evacuated speed/m·s^-1^	1.4~2.8	1.4~2.8	1.4~2.8	1.4~2.8	1.4~2.8	1.4~2.8	1.4~2.8	1.4~2.8

The size and location of the landscape pool and greening in the square are also be confirmed. By setting the scale 1.0 m = 1.5 px, the accuracy of the width, length and position is controlled within 1.0 m.

For the pedestrian parameter setting, the pedestrian normal walking speed range is 1.0 ~ 1.4m/s, the initial speed range is 0.3 ~0.7 m/s, and the pedestrian diameter range is 0.3 ~ 0.4m. The pedestrian speed during evacuation is 1.4 ~ 2.0 times of the normal speed.

Meanwhile, in the simulation calibration, the entry flow of pedestrians is controlled by the separate timetable for every exit *i* in different time periods, and the arrival and departure of pedestrians generally obeys a normal distribution.

#### Calibration of SFM parameters

The SFM has been deeply studied and widely used since it was proposed. Therefore, this study directly calibrated the important parameters of the SFM by referring to the relevant studies and the actual investigation, and designed scenarios to verify the SFM after parameter calibration. The main method was to compare the output value of the model with the actual measured value. If the deviation was too large, the parameters were readjusted. Result of SFM parameters for calibration is shown in Table 5.

**Table 5:** Result of SFM parameters for calibration.

No.	Symbol	Parametric description	Values
1	***τ***	Reaction time	0.15 s
2	***A***_***i***_	Repulsive strength	2.00 m · s^-2^
3	***B***_***i***_	Range of the repulsion	0.20 m
4	***K***	Elasticity coefficient	44,000.00 N · m^-1^
5	***k***	Sliding coefficient	60,000.00 N · m^-1^
6	***A***_***w***_	Repulsive strength of the obstacle *w*	10.00 m · s^-2^
7	***B***_***w***_	Range of repulsion of the obstacle *w*	0.15 m

#### Evaluation of the parameter calibration of the SFM

By comparing the measured data with the output data of the model after parameter calibration, whether the calibration met the requirements was judged. If the requirements were satisfied, the parameter combination obtained was used as the input parameter of the model to analyze the rationality of the model setting. If any requirement was not satisfied, the parameters were recalibrated and evaluated until the verification targets were achieved.

The selected parameter combination was used as the parameter input of the simulation model to select the main scenario for simulation and calculate the evaluation indicator,

$$G=\frac{\left|PM-APM\right|}{PM}$$ (24)

where *G* is the threshold of the indicator, and the absolute value of the relative error in this model; *PM* is the measured data of the indicator; and *APM* is the output data of simulations.

If the threshold satisfied the conditions, it was confirmed that the parameter calibration satisfied the compatibility requirements between the model and the real situation. Otherwise, the parameters were recalibrated until they were subjected to the thresholds. The software AnyLogic was used to extract the corresponding simulation values by designing simulation scenarios similar to the actual. A comparative analysis of the measured and simulated values of the indicators is shown in Table 6. It was seen that the relative absolute errors were less than 5%, indicating that the parameter calibration was acceptable.

**Table 6:** Result of evaluation of parameter calibration.

No.	Facility classification	Facility parameters	Indicator	Measured value	Simulation value	Relative error (%)
1	Stairs	2.00 × 13.00 m	Volume	175 ped/300 s	179 ped/300 s	2.30
2	Stairs	2.00 × 13.00 m	Speed	0.67 m/s	0.69 m/s	3.50
3	Channel	4.20 × 10.00 m	Volume	227 ped/300 s	233 ped/300 s	2.70
4	Channel	4.20 × 10.00 m	Speed	1.31 m/s	1.35 m/s	1.90
5	Subway Platform	1,000.00 m^2^	Speed	0.58 m/s	0.60 m/s	1.60
6	Subway Platform	1,000.00 m^2^	Density	1.52 ped/m^2^	1.49 ped/m^2^	2.10

### Simulation results

**Fig 4:**
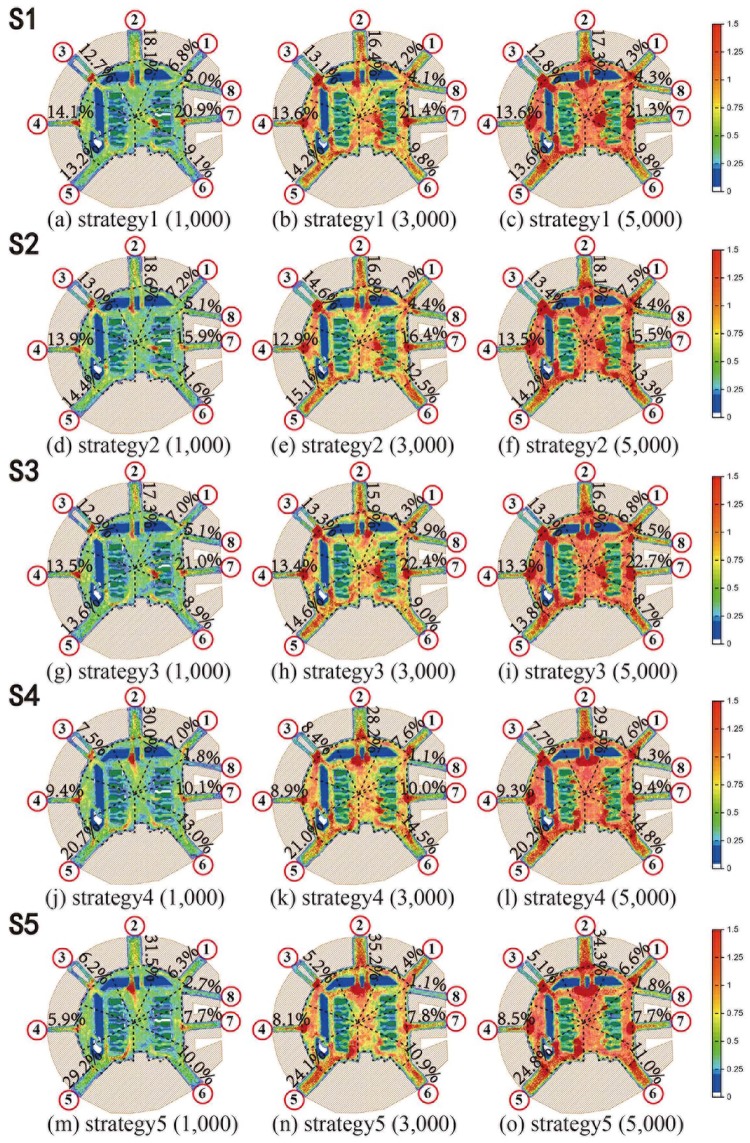
Heatmaps of five evacuation strategies in the LPS.

Simulation experiments, more than 600 simulation runs, the total simulation time were more than 3, 000 minutes obtained. In total, more than 300 sets of simulated evacuation time statistics were obtained, and the utilization rate of every evacuation channel was simulated in more than 300 groups, and more than 200 groups of channel evacuation were simulated in the unit time. Finally, the heat maps of pedestrian evacuation are obtained, as shown in Fig 4. The red color indicates a high-density area, and crowd density is greater than 1.25 m^2^; the blue color represents a low-density area of crowds, and pedestrian density is less than 0.5 m^2^.

In Fig 4, the percentage value indicates the proportion of evacuees in each evacuation experiment, which reflects the influence of different strategies on the evacuation route choice.

Firstly, the performances of same evacuation strategies in different evacuation scenarios are analyzed. Namely, Fig 4(a), 4(b), 4(c), 4(d), 4(e), 4(f), 4(g), 4(h), 4(i), 4(j), 4(k), 4(l), 4(m), 4(n) and 4(o) are compared separately in Horizon. The comparisons show that in all three scenarios, the proportion of each channel in route choices is very close, that is, each evacuation strategy has similar performance in different scenarios with different evacuees.

Secondly, the performances of same evacuation strategies in different evacuation scenarios are analyzed. Namely, Fig 4(a), 4(d), 4(g), 4(j), 4(m), 4(b), 4(e), 4(h), 4(k), 4(n), 4(c), 4(f), 4(i), 4(l) and 4(o) are compared respectively in vertical. The comparisons show that in five experiments of evacuation strategies, the proportion of each channel in route choices is significantly different. When considering channel evacuation capacity (Fig 4(i)), considering route congestion level and channel evacuation capacity (Fig 4(o)), more people were evacuated in No. 2 channel and No. 4 channel. The reason is that No. 2 channel and No.5 channel have larger evacuation capacity. When considering route congestion level (Fig 4(i)), the number of evacuees in each channel is more balanced, compared with Fig 4(c) and Fig 4(f).

Above all, the heatmap in Fig 4 meets the simulation expectation.

## Discussions and conclusions

### Discussions

**Fig 5:**
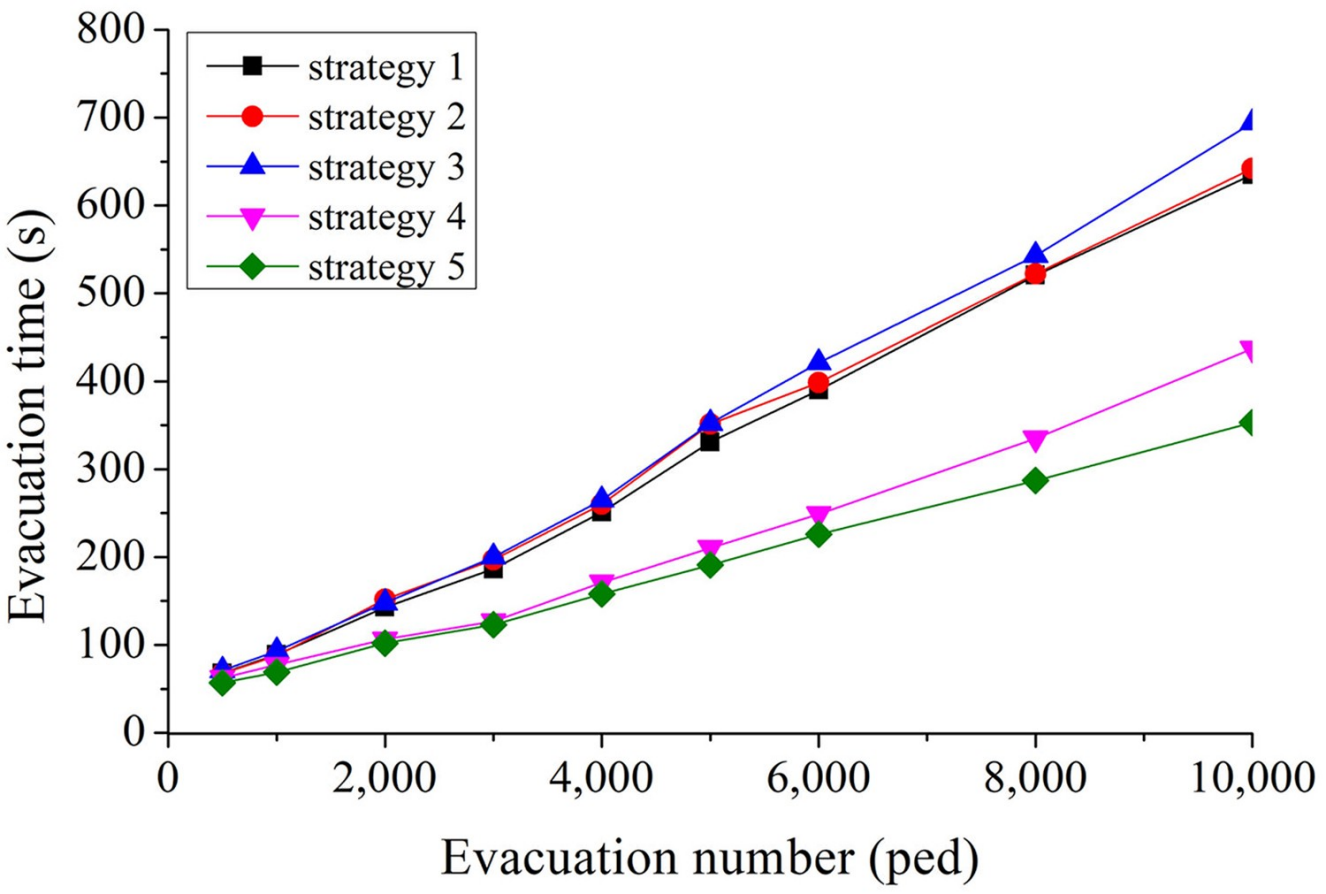
Comparison of evacuation time under five evacuation strategies.

**Fig 6:**
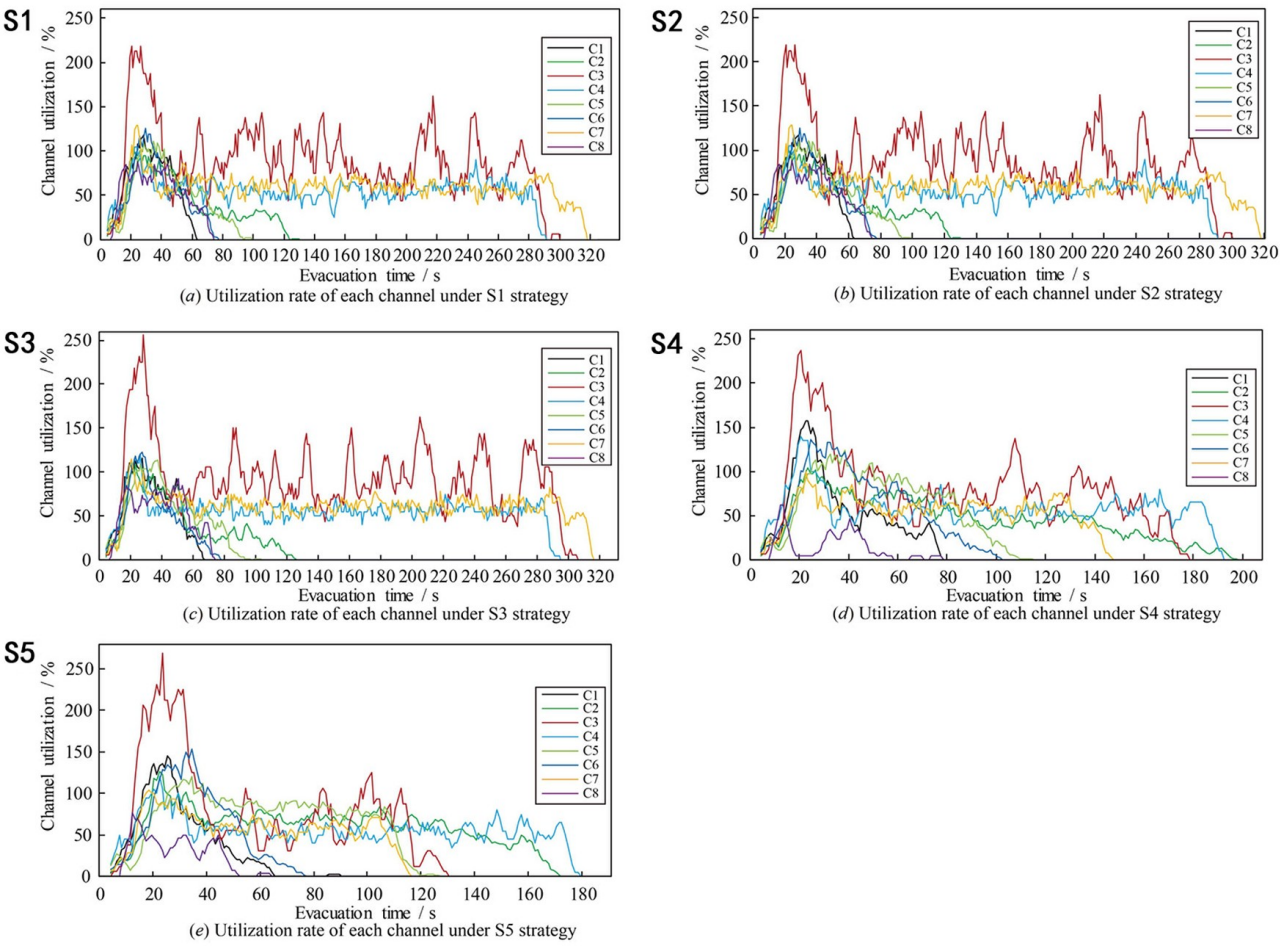
Results of channel utilization rate under five evacuation strategies.

**Fig 7:**
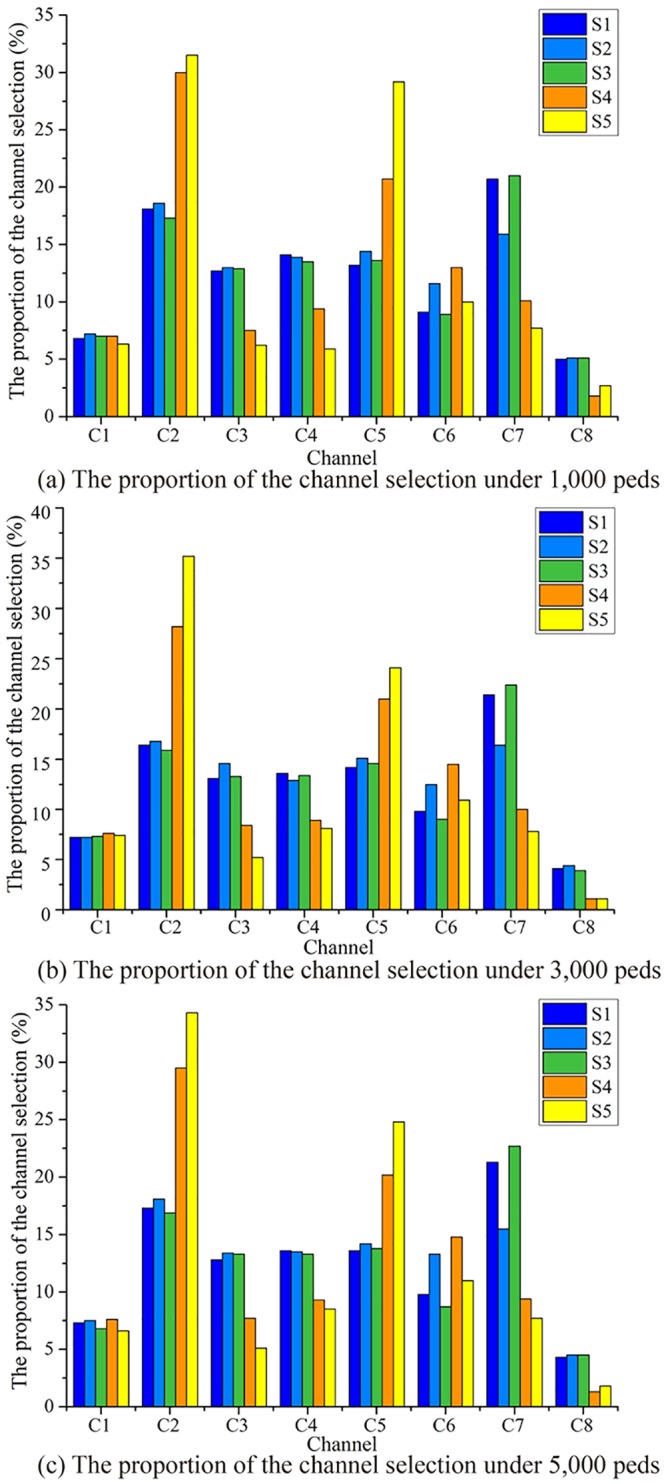
Results of channel selection proportion under five evacuation strategies.

**Fig 8:**
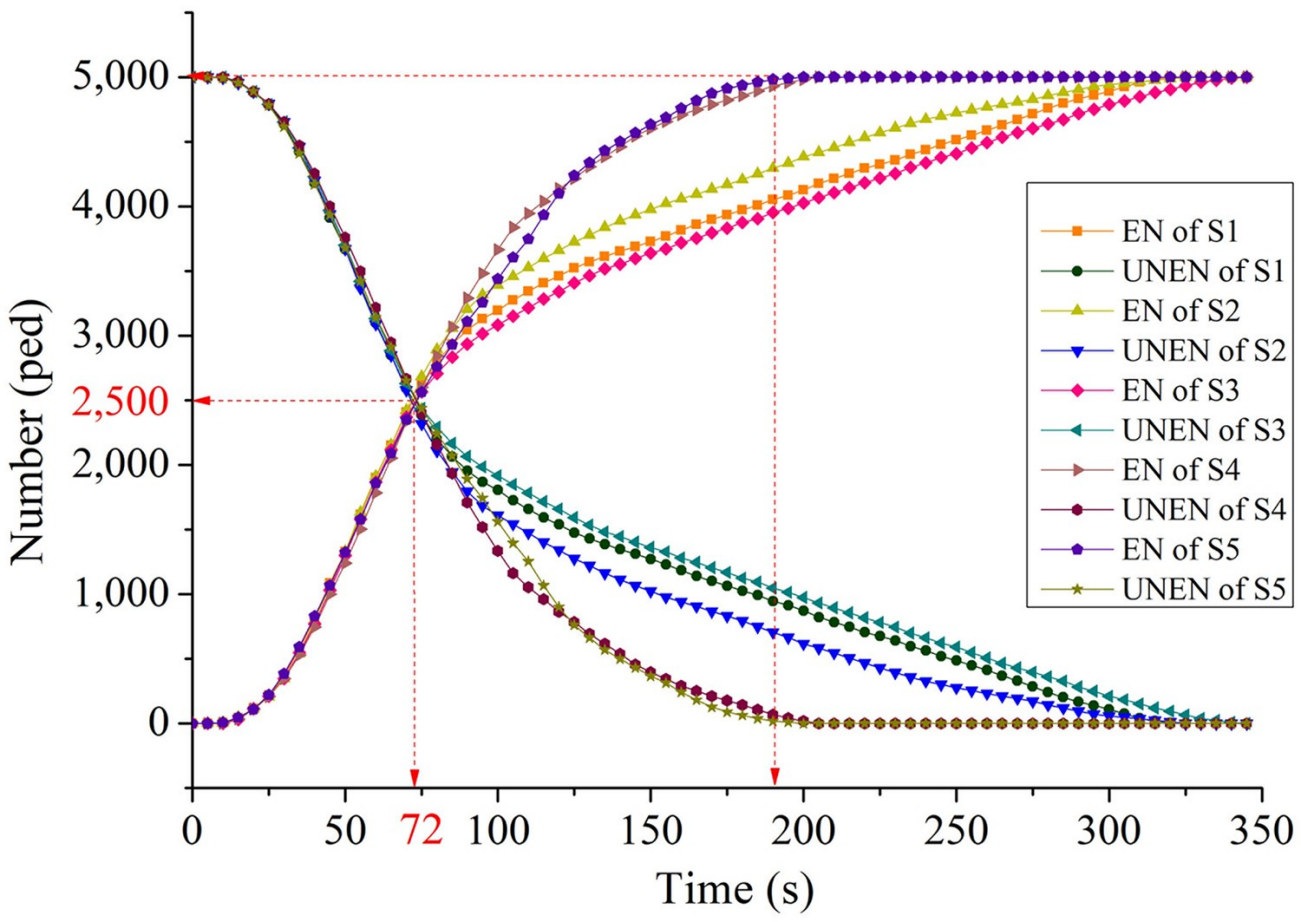
Number of Evacuated and non-evacuated individuals.

1. Evacuation time of pedestrians
  The evacuation time of five evacuation strategies under different evacuation numbers is compared (see Fig 5).
  As shown in Fig 5, the five strategies can be divided into two categories: upper one (S1, S2, and S3), and lower one (S4 and S5). Both S1 and S2 consider the influence of distance on evacuation route choice. The difference between S1 and S2 is, S2 additionally considers the length of channels. Nevertheless, there is no significant difference in the total evacuation time between the two strategies. The main reason is that when the number of evacuators is smaller than the evacuation capacity, the total evacuation time is related to the length of evacuation route. When the number of evacuators exceeds the channel capacity, the total evacuation time mainly exists in the crowded queuing process at the entrance of the channels.
  Both S4 and S5 take into account not only the distance, but also the evacuation capacity (S4), congestion level and evacuation capacity (S5), so that more evacuators will be allocated to channels with larger evacuation capacity or better conditions. These strategies can effectively balance evacuation routes in the square. Compared with S1 and S2, these strategies (S4 and S5) have a shorter evacuation time than those only consider distance.
  In the whole simulation process, the unexpected strategy is S3, which mainly considers congestion level of channels, and its total evacuation time is longer than S1 and S2, in which only distance is considered. S3 considers the congestion level, so that a more balanced evacuator’s distribution is expected. However, when the number of evacuators is small (not exceeding the evacuation capacity), the total evacuation time is more closely related to the route length, and the balanced distribution will not reduce the evacuation time; in the case of oversaturated, balanced distribution but without corresponding evacuation capacity does not make better evacuation time. Compared with S4, it is found that in evacuation allocation, considering the evacuation capacity has more advantages than considering the congestion level, and this further proves the difference between S3 and S1, S2.
2. Utilization of evacuation channel
  In the simulation, the result of channel utilization rate under five evacuation strategies and channel selection proportion are shown in Figs 6 and 7.
  It can be found from Fig 6 that during the whole evacuation process, each channel presents a multi-peak oscillation distribution, and the utilization curve can be divided into three stages. Taking the utilization of No.3 channel as an example, in the first stage (0s ~ 40s), the utilization increases from 0% to 200% of the maximum peak value, and then falls back to less than 100% of the stable value. The curve of this stage is similar to dynamic response curve of second-order underdamped control system under unit step signal input, and the faster the evacuation response, the greater the maximum peak of utilization. In the second stage (41s ~ 280s), the utilization oscillates equally around the average value of 100%, the average amplitude is 40%, and the average oscillation period is 30s. The channels were over-saturated during these two stages. In the third stage (281s ~ the end), the utilization rate of channel under five strategies decreases monotonously to zero.
  From the perspective of different evacuation strategies, it can be found from Fig 6 that in all five strategies, the maximum peak utilization of No.3 channel is the largest, because the width of No.3 channel is the narrowest, and the number of evacuees evacuated in No.3 channel had far exceeded its evacuation capacity, the same result can also be found from Fig 7. The maximum peak utilization of No.8 channel is the smallest, because No.8 channel is very close to No.1 channel, and its length is longer than No.1 channel, so in the evacuation decision-making, the probability of choosing No.1 channel for evacuation is higher than that of choosing No.8 channel, which leads to the lowest utilization No.8 channel. The result of simulation also shows that in S5, compared with other strategies, No.3 channel has the highest maximum peak of utilization, what’s more, Fig 7 also validates the results of Fig 6 further.
3. Evacuation efficiency analysis
  The results show the evacuated number (EN) and non-evacuated number (Non-EN) under the five evacuation route choice strategies. EN of five strategies and Non-EN of five strategies are shown in Fig 8.
  It can be seen from the Fig 8, at the beginning of the evacuation process, when the number of evacuees is less than 2, 500 peds, the fastest evacuation efficiency is S1 and S2. At that time, there is no large congestion queuing phenomenon in the channels. A shorter evacuation route has more advantages, so S1 and S2 have better performance, the evacuation time is 72 seconds.
  As time goes by, when the number of evacuees exceeded 2, 500 peds, pedestrian crowding and queuing appeared at the entrance of each channel. S5 and S4 allocated more pedestrian to channels with larger evacuation capacity, and performed better in total evacuation time. The evacuation time of S5 and S4 is 195 seconds and 200 seconds respectively. For S1 and S2, the total evacuation time is longer, up to 320 seconds, because of the larger queuing time in the channels.
4. Suggestions
  At present, there are four evacuation management measures: increase throughput, prevent blockades, distribute traffic, and limit inflow, which were proposed by Hoogendoorn and Daamen [40, 41].

Based on our research results,

1. For the increase throughput in Tian-yi square, it is not allowed to close any channel, because the total capacity of channels cannot satisfy the demand of evaluation in peak hours.
2. For the prevent blockades in Tian-yi square, the distribution of commercial development, are suitable in the central area of the square, and not suitable in the channels. It can be seen from the simulation results that the capacity of the channel is the most important factor affecting the evacuation efficiency. The commercial utilization and development of any space should not break the capacity of channel, and it should be a basic principle.
3. For the distribute traffic in Tian-yi square, the crowds should be balanced according to the capacity of the channels. If not, the balancing of crowds will have a negative effect in evacuation process.
4. For the limit inflow in Tian-yi square, to complete evacuation in 6 minutes which is from the code for design of metro (GB50157-2013), at peak hours, the total number of passengers in Tian-yi Square should not exceed 5000.

### Conclusions

In this study, based on the classical social force theory, five evacuation methods (considering distance, density and capacity factors respectively) were simulated in Tian-yi square, Ningbo, China, the pedestrian arrival-departure characteristics at large-scale public space was also analyzed. Next, with the increase of the individuals from 500 to 10, 000, the evacuation time of pedestrians and utilization of channels were analyzed in the LPS. The major contributions of this study are summarized as follows,

For the number of evacuees, different evacuation strategies had different effects. In all the evacuation scenarios, the evacuation strategies considering route evacuation capacity (S4), comprehensively considering route evacuation capacity and congestion level (S5) have the best performance, especially S5. The evacuation strategy considering congestion level of routes (S3) has the worst performance, though S3 made a more balanced distribution of evacuees in space, but without considering the corresponding evacuation capacity. The performance of the strategies considering the distance (S1 and S2) are better than S3 and worse than S4 and S5.

For the utilization of channels, the utilization of multi-channel was not balanced, since the five evacuation strategies had different utilization rates for the overall channel. S5 has the most balanced and reasonable utilization rate of the overall channel, to make the shortest evacuation time, and S3 has the opposite. The channel utilization curve in some stages is similar to the dynamic response curve of the second-order control system under the unit step signal input. So in the future we may apply the correlation analysis theory of second-order control system to pedestrian evacuation process.

For the evacuation efficiency, we also found that the strategies considering distance factor have the fastest evacuation responding time, and the strategies considering more factors have the opposite performance. But when there is pedestrian crowding at the channels’ entrance, the evacuation performance of the strategies only considering distance factor deteriorates rapidly. The strategies considering route evacuation capacity (S4), and comprehensively considering route evacuation capacity and congestion level (S5) have the best performance on the evacuation efficiency.

The simulation results enhanced our comprehension of the evacuation strategies related to evacuation route choice and evacuation number. Based on the results, some suggestions regarding planning, engineering, and public advocacy were discussed to increase the safety of large-scale public places in Ningbo, China. As follows, (*a*) Safety campaigns, such as escape drills, could be launched to increase pedestrians’ awareness of law and their awareness of benefits from evacuation; (*b*) It is recommended to enforce crowd management strategies and safety performance among the event venue organizers, such as abnormal crowd detection and tracking, crowd monitoring and early warning; and (*c*) A more effective risk assessment system, as well as a high quality evacuation route choice service, should be developed to make the crowd evacuation process easier and quicker.

However, there are still several problems needing further attention in future work. First, in terms of evacuation scenarios, only pedestrians’ evacuation route choice in normal situation is studied in this paper, while the unexpected event such as fire is ignored. Second, the pedestrian heterogeneous characteristics [42] such as age, gender, and partner relationship should also be considered as the influencing factors of route choice behavior. These factors could also be further examined using Bayesian approach [43, 44]. In addition, the extension of this study would be to examine the unobserved heterogeneity across crowds and evacuation route optimization, the full Bayesian random parameters multivariate Tobit model [44] and multiple-objective genetic algorithm [45] will be a good solution method for the above issues. Recent works [1; 15, 17, 18, 22, 23] only provided the framework of pedestrian route choice behavior in escape panic. Under this framework, external information parameters effects on crowd dynamics and route choice can be estimated. These limitations will be improved by a more elaborate modeling approach and a strict calibration procedure in our future work.

## Supporting information

S1 Data Data used for figures. (XLSX)
